# Comparison of Lumbar Fusion With and Without Interbody Fusion for Lumbar Stenosis Using Patient-Reported Outcomes Measurement Information System (PROMIS) Computer Adaptive Testing (CAT)

**DOI:** 10.7759/cureus.23467

**Published:** 2022-03-24

**Authors:** Michael McCarthy, Peter R Swiatek, Anastasios G Roumeliotis, Erik Gerlach, Jeffery Kim, Barrett S Boody, Melissa Shauver, Wellington K Hsu, Alpesh A Patel

**Affiliations:** 1 Orthopaedics, Spine Surgery, Hospital for Special Surgery, New York, USA; 2 Orthopaedic Surgery, Northwestern University Feinberg School of Medicine, Chicago, USA; 3 Orthopaedic Surgery, Indiana Spine Health, Indianapolis, USA; 4 Orthopaedic Surgery, Northwestern Memorial Hospital, Chicago, USA

**Keywords:** spinal stenosis, spondylolisthesis, patient reported outcomes measurement, posterior spinal fusion, interbody fusion

## Abstract

Study design

This was a retrospective analysis of patient-reported outcomes across a two-year period.

Summary of background data

Patients suffering from lumbar stenosis may experience low back pain, neurogenic claudication, and weakness. Patients can benefit from surgical intervention, including decompression with or without fusion. However, the superiority of any single fusion construct remains controversial.

Objective

The goal of this study was to compare Patient-Reported Outcomes Measurement Information System (PROMIS®) Computer Adaptive Testing (CAT) measures in patients with lumbar spinal stenosis treated surgically with lumbar decompression and fusion with or without interbody fusion.

Methods

A retrospective review of patients with lumbar stenosis undergoing lumbar decompression and one-level fusion was performed. PROMIS® CAT Physical Function (PF) and Pain Interference (PI) assessments were administered using a web-based platform pre and postoperatively.

Results

Sixty patients with lumbar stenosis undergoing one-level lumbar fusion were identified. Twenty-seven patients underwent posterior lumbar fusion (PSF) alone and 33 underwent one-level lumbar interbody fusion (IF). Patients undergoing IF had better absolute PF scores compared to patients undergoing PSF at one-year postoperatively (48.9 v 41.6, p=0.002) and greater relative improvement in PF scores from baseline at one-year postoperatively (D13.6 v D8.6, p=0.02). A subgroup analysis of patients undergoing TLIF v PSF showed better absolute PF scores at the one-year follow-up in the TLIF group (47.1 v 42.3, p=0.04). No differences were found in PI scores at any time point between the PSF and IF groups. Patients undergoing IF had significantly shorter hospital stays (2.5 v 3.3 days, p=0.02) compared to patients undergoing PSF.

Conclusions

Patients with lumbar spinal stenosis treated with one-level IF reported higher absolute PF scores and experienced greater relative improvement in PF scores from baseline at one-year follow-up compared to patients treated with PSF alone. Additionally, IF is associated with a decreased length of hospital stay as compared to PSF.

## Introduction

Lumbar stenosis is caused by a variety of pathologies resulting in narrowing of the spinal canal and subsequent compression of neurologic elements. Clinical symptoms can manifest as lower back pain, radiculopathy, and neurogenic claudication. The Spine Patients Outcomes Research Trial (SPORT) compared operative versus medical management of lumbar stenosis and demonstrated the superiority of surgery compared to nonoperative care at the two and four-year follow-ups, especially when radicular symptoms prevailed [1]. These results, in addition to new techniques and implant technologies, support the operative management of lumbar stenosis when conservative measures fail [2].

Laminectomy remains the gold standard of surgical treatment for lumbar stenosis and is associated with good clinical outcomes [3]. Patients with instability, most commonly degenerative spondylolisthesis, are often additionally treated with spinal fusion. In an effort to improve fusion rates, spine surgeons are increasingly utilizing interbody fusion; however, interbody fusion is associated with an increased risk of complication and additional costs of instrumentation [4-5]. Supporters of the interbody devices cite evidence that demonstrates higher fusion rates with interbody cage placement [6]. Critics, on the other hand, point to recent literature showing no significant difference in patient outcomes between those treated with interbody fusion versus posterior lateral fusion alone [7]. Overall, there is limited evidence for any single-level fusion construct, and decisions are ultimately based on patient anatomy, spinal pathology, and a surgeon’s skill set [8].

The purpose of this study is to compare patient-reported outcomes utilizing the Patient Reported Outcomes Measurement Information System (PROMIS®) Computer Adaptive Testing (CAT) for lumbar stenosis patients undergoing surgical decompression and one-level lumbar interbody fusion to those with posterolateral fusion alone.

## Materials and methods

This study was reviewed and approved by our institutional review board at Northwestern University (IRB Number: NU STU00205674). Data were retrospectively identified from a prospectively collected database of consecutive patients treated by any of three fellowship-trained spine surgeons at a single academic institution between 2013 and 2017. Patient-reported outcomes were obtained using PROMIS® Physical Function (PF) and Pain Interference (PI) via computer-adaptive tests (CATs). Patients were between 24 and 77 years of age and presented with radiographic and clinical findings consistent with lumbar spinal stenosis. Inclusion criteria included patients with lumbar decompression and one-level fusion, with or without interbody fusion. Approaches for the placement of the interbody fusion device were left to surgeon discretion and included anterior, lateral, and transforaminal approaches. A minimum of one-year follow-up was required. Surgical technique was based on patient anatomy, spinal pathology, and a surgeon’s skill set. Patients with a previous history of scoliosis, lumbar surgery, trauma, infection, and osseous neoplasms were excluded. Patients with multilevel fusion were also excluded.

Primary outcome measures were the PROMIS® Physical Function (PF) and Pain Interference (PI) scores. PROMIS® and legacy measures were administered preoperatively and postoperatively at six weeks, three months, one year, and two years. Initial assessments were conducted in the clinic using a tablet and follow-up assessments were administered via the internet or over the phone. Secondary outcome measures included perioperative outcomes such as blood loss, hospital length of stay, operating time, and reoperation rate. The PROMIS® Physical Function Item Bank is composed of 121 potential questions and provides patients’ perceived capabilities of physical activity. The PROMIS® Pain Interference Item Bank consists of 41 potential items and measures the role pain plays in daily activities.

Descriptive statistics were calculated for patient demographics and outcomes at baseline. PROMIS® CAT scores were directly exported from the Assessment CenterSM system [9]. Participants with data from assessments made up to two years postoperatively were included in the analysis (i.e., preoperative, six weeks, three months, one year, and two years). Continuous variables were represented as a mean and standard deviation. Categorical variables were represented as numbers and percentages of the total. The resulting data were stratified by posterior spinal fusion alone (PSF) and interbody fusion (IF). A subgroup analysis of patients undergoing transforaminal interbody fusion (TLIF) was also completed. The results were graphically analyzed to display changes in main outcomes and the correlation structure of repeated measurements. The statistical significance of changes between each assessment point was evaluated using single student t-tests.

## Results

A total of 60 patients met inclusion criteria and had completed the baseline, six-week, three-month and one-year assessments. The majority of these patients (51/60) also completed two-year assessments. Just short of half the group underwent PSF (27/60) versus IF (33/60). The majority of patients were female in both the PSF (67%, 18/27) and IF (55%, 18/33) groups, and the mean age of the PSF and IF groups were 66.6 and 54.0, respectively (Table 1).

**Table 1:** Demographic data of patients undergoing PSF versus IF PSF: posterior lumbar fusion; IF: interbody fusion

	PSF N=27	IF N=33	p-value
Age, yrs, mean (SD)	66.6 (5.2)	54.0 (14.8)	<0.001
Body Mass Index (BMI), mean (SD)	29.5 (5.2)	31.1 (7.1)	0.32
Gender, n (%)			
Male	9 (33%)	15 (45%)	0.34
Female	18 (67%)	18 (55%)	

Of the total 60 patients, 55 were diagnosed with spondylolisthesis, with each participant subsequently undergoing one-level fusion. The IF group was composed of four anterior interbody fusions (ALIF), four direct lateral interbody fusions (DLIF), and 25 transforaminal interbody fusions (TLIF). All patients underwent posterior instrumentation. In the TLIF group, 12 patients (48%) underwent a minimally invasive approach, which at our institution is operating through tubes, compared to the standard open posterior approach. Patients undergoing IF had a significantly shorter hospital stay (2.5 days) compared to patients undergoing PSF (3.3 days) (p=0.02) (Table 2). There was no significant difference in blood loss, operative time, or reoperation rate between PSF and the IF groups.

**Table 2:** Operative and recovery characteristics of the PSF and IF groups PSF: posterior lumbar fusion; IF: interbody fusion

	PSF N=27	IF N=33	p-value
Blood loss, mL, mean (SD)	188.3 (174.6)	127.3 (96.1)	0.09
Hospital length of stay (LOS), days mean (SD)	3.3 (1.3)	2.5 (1.3)	0.02
Operating time, mins, mean (SD)	133.6 (39.5)	145.9 (30.3)	0.18
Reoperations, n (%)	4 (15%)	2 (6%)	0.39

**Figure 1:**
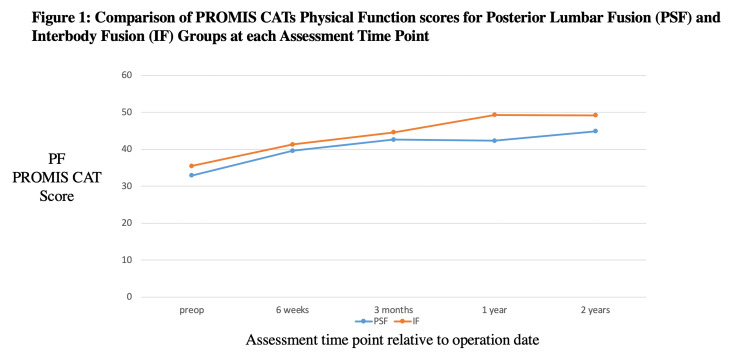
Comparison of PROMIS CATs physical function scores for PSF and IF groups at each assessment time point PSF: posterior lumbar fusion; IF: interbody fusion

Overall, at the baseline assessment, pain interference (PI) and pain function (PF) scores were 14.9 and 15.6 points worse than the general population mean, indicating a greater than average impairment among our study population. The PROMIS® CAT assessments showed variability of patient-reported PF scores between the PSF and IF groups. Preoperatively, PF scores were not significantly different, at 33.1 and 35.4 for the PSF and IF groups, respectively (p=0.11). PF scores at one year postoperatively were 41.6 and 48.9 for the PSF and IF groups, respectively (p=0.002) (Table 3). There was no significant difference in PF scores at other time points (Figure 1).

**Table 3:** Comparison of PROMIS CAT Pain Interference (PI) and Physical Function (PF) scores in the PSF and IF groups across time (preoperative, six weeks, three months, one year, and two years) PSF: posterior lumbar fusion; IF: interbody fusion

	PSF PROMIS CAT mean score (SD)	IF PROMIS CAT mean score (SD)	p-value
Preoperative				n=27	n=33	
Pain Interference (PI)	65.3 (5.3)	64.5 (8.6)	0.66
Physical Function (PF)	33.1 (5.2)	35.4 (5.6)	0.11
6 weeks	n=27	n=33	
Pain Interference (PI)	54.1 (10.1)	58.1 (8.4)	0.10
Physical Function (PF)	39.5 (7.8)	41.0 (7.0)	0.43
3 months	n=27	n=32	
Pain Interference (PI)	53.3 (10.1)	55.6 (8.9)	0.34
Physical Function (PF)	42.5 (8.2)	44.0 (7.0)	0.45
1 year	n=27	n=33	
Pain Interference (PI)	53.3 (10.9)	51.7 (8.2)	0.56
Physical Function (PF)	41.6 (9.7)	48.9 (7.2)	0.002
2 years	n=22	n=29	
Pain Interference (PI)	53.1 (10.4)	49.6 (9.6)	0.21
Physical Function (PF)	44.6 (11.8)	49.1 (9.6)	0.15

There was no significant difference in PI scores between the PSF and IF groups at any time point. To better assess the relative change in preoperative PF scores for the PSF and IF groups, a delta (D) analysis was performed to better compare the improvements associated with each intervention. The calculated differences in patient-reported outcomes between the PSF and IF groups relative to baseline preoperative assessment (delta scores) are demonstrated in Table 4.

**Table 4:** Comparison of the mean delta (D) of PROMIS CAT Pain Interference (PI) and Physical Function (PF) scores from preoperative to six weeks, three months, one year, and two years in the PSF and IF subgroups PSF: posterior lumbar fusion; IF: interbody fusion

	PSF Mean D score (SD)	IF Mean D score (SD)	p-value
PROMIS CAT			
Pain Interference (PI)			
D preoperative to 6 weeks	-11.2 (10.0)	-6.3 (9.3)	0.06
D preoperative to 3 months	-12.0 (10.3)	-9.7 (10.5)	0.39
D preoperative to 1 year	-12.1 (11.2)	-12.7 (10.4)	0.83
D preoperative to 2 years	-13.0 (12.0)	-15.4 (9.1)	0.41
Physical Function (PF)			
D preoperative to 6 weeks	6.4 (7.9)	5.7 (8.0)	0.71
D preoperative to 3 months	9.4 (8.4)	8.6 (6.7)	0.69
D preoperative to 1 year	8.6 (9.3)	13.6 (7.0)	0.02
D preoperative to 2 years	11.6 (11.8)	14.4 (9.7)	0.36

Our analysis demonstrates a statistically significant increase in PF scores at one year when compared to the preoperative baseline scores. At one year, the IF group presented with a greater increase in PF score when compared to the PSF group (D13.7 versus D9.4, p=0.02) (D = delta). This indicates that patients who underwent IF, compared to PSF, had a greater increase in physical function compared to preoperative status at one year. The significance of this difference, however, was lost at the two-year follow-up, with PF scores for the PSF and IF groups reported as 11.6 and 14.4 (p=0.36), respectively. There were no significant differences between groups in the delta PI scores at six weeks, three months, one year, and two years postoperatively when compared to the preoperative baseline PI scores.

Of the patients who underwent IF, the majority (25/33) underwent transforaminal lumbar interbody fusion (TLIF). Patients undergoing TLIF experienced statistically better absolute PF scores at one year compared to the PSF group (47.6 vs. 41.6, p=0.01) (Table 5).

**Table 5:** Comparison of PROMIS CAT Pain Interference (PI) and Physical Function (PF) scores in the PSF and TLIF groups across time (preoperative, six weeks, three months, one year, and two years) PSF: posterior lumbar fusion; IF: interbody fusion

	PSF PROMIS CAT mean score (SD)	TLIF PROMIS CAT mean score (SD)	p-value
Preoperative				n=27	n=25	
Pain Interference (PI)	65.3 (5.3)	64.4 (9.5)	0.68
Physical Function (PF)	33.1 (5.2)	35.3 (5.7)	0.15
6 weeks	n=27	n=25	
Pain Interference (PI)	54.1 (10.1)	58.6 (8.8)	0.10
Physical Function (PF)	39.5 (7.8)	40.5 (7.5)	0.64
3 months	n=27	n=24	
Pain Interference (PI)	53.3 (10.1)	56.7 (8.6)	0.21
Physical Function (PF)	42.5 (8.2)	43.6 (7.3)	0.60
1 year	n=27	n=25	
Pain Interference (PI)	53.3 (10.9)	52.8 (8.0)	0.88
Physical Function (PF)	41.6 (9.7)	47.6 (6.3)	0.01
2 years	n=22	n=22	
Pain Interference (PI)	53.1 (10.4)	49.7 (9.6)	0.26
Physical Function (PF)	44.6 (11.8)	48.0 (7.1)	0.27

There was no difference in PF scores preoperatively, at six weeks, three months, or two years. There was no difference in PI scores at any time period. Additionally, the subset analysis showed an appreciable difference in length of hospital stay between the TLIF and PSF group (2.2 days vs. 3.3 days, respectively, p = 0.0003) (Table 6).

**Table 6:** Operative and recovery characteristics of the PSF and TLIF groups PSF: posterior lumbar fusion; IF: interbody fusion

	PSF N=27	TLIF N=25	p-value
Blood loss, mL, mean (SD)	188.3 (174.6)	136.0 (102.6)	0.19
Hospital length of stay (LOS), days mean (SD)	3.3 (1.3)	2.2 (0.80)	0.0003
Operating time, mins, mean (SD)	133.6 (39.5)	140.0 (25.0)	0.50
Reoperations, n (%)	4 (15%)	2 (8%)	0.44

There was no significant difference in blood loss, operative time, or reoperation rate between PSF and TLIF groups.

## Discussion

The role of surgical management of lumbar stenosis is becoming more prominent, especially in clinically symptomatic patients with radiographic stenosis refractory to conservative measures [10]. Laminectomy serves as the primary surgical treatment for lumbar stenosis, with recent trends favoring the addition of spinal fusion, specifically with interbody devices. Currently, there is a limited consensus of the standard of care regarding surgical techniques for spinal fusion and conflicting literature with respect to the incremental benefit of interbody arthrodesis [11-12].

Overall, our results show improved outcomes in patients with lumbar stenosis who were treated with posterior spinal fusion or interbody fusion as measured by PROMIS® PF and PI scores. Patient-reported outcome scores reached the minimal clinically important difference (MCID) threshold (5 points) at all time points of the study. Our results demonstrate statistically better absolute PROMIS® PF outcomes among patients with lumbar stenosis who were treated with one-level lumbar IF compared to those treated with one-level PSF at one year. When considering the improvement from baseline physical function before surgery to after surgery (i.e., delta score), our results further showed that IF was associated with a greater improvement in PF scores at one year postoperatively compared to PSF. Although PF scores for IF patients remained superior to those of PSF patients at two years, the magnitude was not statistically significant. A subgroup analysis of the patients undergoing TLIF versus PSF demonstrated that TLIF patients demonstrated statistically better PF scores at one year postoperatively.

Regarding the secondary outcomes of the study, our results show that patients undergoing PSF had an appreciable increase in length of hospital stay when compared to patients undergoing IF or TLIF. In the era of bundled payments and increased pressure of cost containment, a decreased length of stay could present an opportunity to decrease health care expenditure. A recent multivariate analysis of over 350,000 patients undergoing spinal fusion surgery identified length of hospital stay as one of the key drivers of cost after spinal fusion surgery [13]. Interestingly, our analysis showed no difference in blood loss or operative time between PSF and IF or TLIF groups, which is contrary to findings from previous studies [7,14].

An overwhelming majority of patients (55/60, 92 %) in our study were noted to have degenerative spondylolisthesis associated with their lumbar spinal stenosis. Within this subset of patients, studies have supported surgical intervention with decompression surgery and fusion in an attempt to avoid potential iatrogenic instability, restenosis, and pseudoarthrosis [15-17]. Studies by Ghogawala et al. demonstrate the superiority of fusion compared to decompression alone for spondylolisthesis, further establishing the newfound role of fusion within this population of patients [18]. While spondylolisthesis in the setting of stenosis supports the indication for fusion with and without IF, there remains controversy regarding the incremental benefit of fusion compared to decompression alone. 

A major strength of the paper is the prospective use of PROMIS® patient-reported outcome measures. PROMIS® is an adaptive and responsive assessment tool that measures patient-related health status. PROMIS® CATs can offer a brief yet precise assessment in a range of domains of self-reported health [19]. Compared to historical measures used in the assessment of outcome in lumbar spine surgery, such as the Oswestry Disability Index (ODI), PROMIS demonstrates greater validity with smaller floor and ceiling limitations [20]. These improved characteristics of PROMIS may partially explain the study’s findings. For example, a recent meta-analysis of 721 patients claims that there is no difference in functional outcome following posterior fusion alone compared to that with interbody fusion [21]. Similarly, Cheng et al. found that although IF provides enhanced fusion rates, there is no statistically significant difference in functional outcome between IF and PSF [22]. Both of these studies utilized traditional outcomes measures, including ODI and visual analog scale (VAS) scores. By using PROMIS® CAT scores, our study presents a new, and perhaps more comprehensive, approach to comparing the impact of IF versus PSF on patient outcomes.

There are a number of limitations to the study. Measurements did not include the degree of preoperative spinal instability, preventing analysis of its impact on patient outcomes. The patients who met the inclusion criteria of the study were not randomized and the decision for IF or PSF was left to the discretion of the surgeon, with the patient characteristics, anatomy, and spinal pathology in mind. Regarding patient anatomy and pathology, surgeons should determine whether the use of an interbody device could provide structural advantages compared to decompression and PLF. For example, surgeons may choose to use an interbody device to provide greater indirect decompression of the foramina and improve patient lordosis [23]. Regarding patient characteristics, surgeons must consider patient age and bone quality when deciding whether the use of an interbody device is appropriate. For example, younger patients typically have more robust bone density and thus their spinal column can support interbody devices. Older patients, on the other hand, are more likely to have osteopenia or osteoporosis, which is associated with a greater risk of endplate collapse around an interbody device [24-25]. Moreover, the PSF group was older than the IF group. As discussed, this is an inherent limitation to the study, as the characteristics of the IF construct may be more favorable in a younger population compared to their older counterparts. According to a literature review by Epstein, overlapping comorbidities accompanying patients older than 65 greatly increase the risk and complication rates associated with spinal surgery [26]. This finding, coupled with the increased age of our PSF group, may possibly present confounding variables and influence the identified PF differences.

## Conclusions

In conclusion, this study demonstrated that patients with lumbar stenosis with spondylolisthesis treated with decompression and one-level interbody fusion had a greater improvement in physical function scores at one year compared to those treated with posterior spinal fusion alone. The improvement from baseline between the IF and PSF groups, although present at the two-year follow-up, was not statistically significant. This suggests that IF may provide superior outcomes to PSF in the short term but the long-term benefits of IF are less clear. Furthermore, IF is associated with decreased length of hospital stay compared to PSF. Additional research investigating the difference in patient-reported outcomes for IF and PSF patients will help further elucidate the potential benefits of IF versus PSF.
